# Innovation in biosafety oversight: The Harvard Catalyst Common Reciprocal IBC Reliance Authorization Agreement

**DOI:** 10.1017/cts.2019.405

**Published:** 2020-02-26

**Authors:** Rebecca Caruso, Theodore Myatt, Barbara E. Bierer

**Affiliations:** 1 Harvard Medical School, Boston, MA, USA; 2 Brigham and Women’s Hospital, Boston, MA, USA; 3 Division of Global Health Equity, Department of Medicine, Brigham and Women’s Hospital, Boston, MA, USA

**Keywords:** Biosafety, Institutional Biosafety Committee, compliance, recombinant DNA, multi-institutional reliance

## Abstract

Increasingly, basic, translational, and clinical research has become more collaborative, resulting in multi-institutional studies that involve common approaches to a central question. For multi-institutional projects that involve recombinant or synthetic nucleic acids, Institutional Biosafety Committee (IBC) review is generally required at each separate site. Duplicative review may result in both administrative costs and delays, without evidence of increased safety or protections, and investigator frustration. To address these inefficiencies, IBC leaders drafted a collaborative IBC Reliance Authorization Agreement. The Agreement allows one or more institutions to cede IBC review to a reviewing IBC that accepts the responsibility. The ability to cede IBC review, and the ability to rely on one decision on behalf of all collaborating institutions for a given protocol, removes delays in approval of multi-center protocols, and collaborating principal investigators are able to focus on research rather than administrative tasks. In the process, we found promotion of this collaborative model led to stronger connections among institutions and among IBC members. The requirement for IBC member representation from the local community, however, limits its broader dissemination; we make several recommendations to mitigate this challenge.

## Introduction

The National Institutes of Health (NIH) Guidelines for Research Involving Recombinant or Synthetic Nucleic Acid Molecules (“NIH Guidelines”) [1] require all research involving recombinant or synthetic nucleic acids (“rDNA”) to be reviewed and approved by an Institutional Biosafety Committee (IBC), if that research is conducted at institutions that receive funding from federal agencies that include the NIH Guidelines in their Terms and Conditions for funding. While the NIH Guidelines do not have the force of a regulation, institutions that receive any federal funding for research involving rDNA must comply with the Guidelines for all rDNA research at the institution, even if a specific project is not federally funded.

In the mid 1970s, the potential biohazards of rDNA research led individuals representing legal, community, regulatory, and scientific perspectives to draft voluntary guidelines to ensure the safety of rDNA technology [2]. Following the conference, in 1976, the NIH developed the NIH Guidelines to address ethical and societal concerns about rDNA research, including public health, safety, and the potential environmental impact of the research. While a number of federal bills were later introduced with the specific aim to regulate rDNA research, none passed, in part due to the success of the framework that the NIH Guidelines provided [3].

While there are no federal regulations for rDNA research, a number of municipalities have established local ordinances that require compliance with the NIH Guidelines regardless of the source of research funding. Like many municipalities, both Cambridge and Boston have introduced regulations that require institutions to register both rDNA and biological materials and, additionally, that require permit holders to (1) allow reasonable inspections upon initial application and whenever an amendment to ongoing research is proposed; (2) establish an IBC and medical surveillance program; (3) develop a health and safety manual and safety training program; (4) report all incidents to the city; and (5) provide an annual report [4–7].

Since the development of the NIH Guidelines and local rDNA regulation, the utility of rDNA as a tool for basic and translational research has grown significantly [8]. In addition, basic, translational, and clinical research has become more collaborative over time, resulting in multi-institutional studies that involve multiple investigators employing complementary or common approaches to a central question. Consequently, the number and complexity of research protocols requiring IBC review has steadily grown [9]. The IBC is constituted to review and approve all studies involving rDNA of animals or humans prior to study initiation. In addition, it is considered best practice – and often an institutional requirement – to review biological agents for their safe use. The regulatory burden is especially time-consuming for human subject gene-transfer protocols that require review by both an Institutional Review Board (IRB) and IBC and that have additional reporting requirements to the US Food and Drug Administration (FDA). Because the NIH no longer independently reviews gene therapy clinical trials [10], the role of local IBC review has risen in importance.

The NIH Guidelines require every IBC to empanel “at least two members [who] shall not be affiliated with the institution (apart from their membership on the Institutional Biosafety Committee) and who represent the interest of the surrounding community with respect to health and protection of the environment” [10]. As a consequence, collaborative studies involving multiple institutions are required to be reviewed by multiple IBCs, resulting in administrative delays and investigator frustration in the absence of published evidence of increased safety or protections. The expectation of multiple IBC reviews contrasts with IRB review of human subjects research wherein one institution may defer review to another institution.

We sought to develop a model agreement to support single IBC (sIBC) review for multi-institutional research, with the goals of reducing administrative burden and of achieving efficiencies of review for multisite clinical or laboratory research involving rDNA and/or other materials reviewed by the IBCs (e.g., biological agents), not unlike the single IRB (sIRB) model for human subject research review. Over the course of approximately 24 months, the institutions developed a reliance agreement to allow for a consolidated sIBC review for any multi-center clinical trial or collaborative laboratory research study that involved the signatory institutions.

## Methods

### Framework of the Master IBC Reliance Agreement

The Harvard Clinical and Translational Science Center (Harvard Catalyst), funded by the NIH Clinical and Translational Science Awards Program, is focused on improving clinical and translational research, including regulatory processes and operations. We previously developed a legal framework for reliance among IRBs, with the goal of improving IRB review efficiency for multi-institutional studies [11,12]. The Harvard Catalyst Master Reciprocal Common IRB Reliance Agreement has evolved into SMART IRB, a national platform^1^ to enable IRB reliance and advance collaborative research across the nation [12,13].

With the background and experience of developing a prototype sIRB agreement, a Harvard Catalyst IBC Working Group representing 16 legally independent 501(c) (3) institutions (Table 1) and 5 IBCs was assembled to develop a sIBC agreement. It is important to note that the signatory institutions of this agreement have a history of collaboration, including the Harvard Medical School IBC having authority for IBC oversight of nine other Harvard-affiliated institutions. In many cases, IBC membership is composed of faculty that hold joint appointments at both Harvard and one of the deferring (relying) institutions. The framework for the sIBC agreement, however, is more expansive than prior agreements between Harvard and other individual institutions and was developed through discussions with IBC leadership and the institutional attorneys of Harvard and its affiliated hospitals as well as outside counsel. Early in the process, the conceptual approach was discussed with representatives of NIH Office of Science Policy (OSP) to elicit feedback on a proposed framework compliant with the NIH Guidelines. The guidance OSP provided confirmed that the Agreement would have to allow for review by an appropriately constituted IBC (i.e., unaffiliated membership from the local community) with authority to review at the given institution [10]. Parallel to the Harvard Catalyst IBC Working Group process, the NIH OSP released guidance documents for facilities exploring the concept of a reviewing/relying relationship [15]. These documents included frequently asked questions on externally administered IBCs and helped guide the process. The Harvard Catalyst IBC Working Group considered these in formalizing the IBC Agreement [14].

**Table 1.** Signatories of the Institutional Biosafety Committee (IBC) agreement

Boston Children’s Hospital
Dana-Farber Cancer Institute
Forsyth Dental Infirmary for Children d/b/a The Forsyth Institute
Harvard Faculty of Arts and Sciences
Harvard Medical School
Harvard School of Dental Medicine
Harvard T.H. Chan School of Public Health
Beth Israel Deaconess Medical Center, Inc.
Joslin Diabetes Center
Massachusetts Eye and Ear Infirmary
Schepens Eye Research Institute
Partners HealthCare System, Inc.
The Brigham and Women’s Hospital, Inc.
The General Hospital Corporation d/b/a The Massachusetts General Hospital
The McLean Hospital Corporation
The Spalding Rehabilitation Hospital Corporation

The leadership team met biweekly for 2 years to determine the standards for review, the obligations and responsibilities of both the reviewing IBC and relying institution(s), and the authority for assurance, compliance, and reporting. The draft agreement was reviewed by counsel at each of the signatory institutions, revised, and re-reviewed. When the draft agreement was acceptable to counsel, the institutional official at each institution reviewed the final agreement prior to signature.

### Agreement Characteristics

The resulting Master IBC Reliance Agreement (“IBC Agreement”) [14] provides a flexible and replicable framework for IBC reliance that can be adapted by other academic medical centers and research institutions that, given the current NIH Guidelines [10], share geographic location. The requirement that IBC membership includes two members “of the surrounding community” [10] has been interpreted narrowly to imply that the community members must be residents of that community; the IBC Agreement, therefore, is currently limited in its reach to local institutions.

**Fig. 1.**
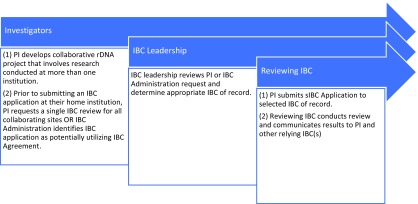
Harvard catalyst reliance model Institutional Biosafety Committee (IBC) cede review process. The process for IBC reliance involves not only the IBC but also institutional and IBC leadership and the involved investigators. PI, Principal Investigator; sIBC, single IBC.

The Master IBC Agreement allows for sIBC review across 16 institutions in Boston and Cambridge, MA. The initial signatories are listed in Table 1. The IBC Agreement specifies a process whereby the Principal Investigator (PI) or IBC administrator can propose that an institutional IBC review on behalf of other institutions or, alternatively, cede review to the IBC of another institution for a specified project. In order to request ceded review, investigators must complete and submit a Cede Review Form prior to submitting their IBC application. Each participating IBC makes the decision on a protocol-by-protocol basis whether to rely on the review of another IBC (to cede the review) or to conduct its own review. The IBC Agreement does not require or prescribe reliance for all protocols; each institution determines the appropriateness of reliance on a protocol-by-protocol basis. The process for ceding review is outlined in Fig. 1.

The bulk of the IBC Agreement is devoted to defining roles and responsibilities of the Reviewing IBC and the Relying Institution. Section IV of the NIH Guidelines (2019) is devoted to the roles and responsibilities of the institution, the IBC, the Biological Safety Officer (BSO), and the PI. The IBC, in particular, has numerous responsibilities, including assessing the containment levels, facilities, and procedures involved in the proposed research. The IBC is also required to review and screen the expertise of personnel listed on each project to ensure appropriate education and training. The sIBC Agreement delineates the responsibilities that are shared or partitioned between the Reviewing IBC and the Relying Institution. The reviewing IBC has responsibility for the elements of the IBC protocol review including review of the application, access to minutes, approval notification and updates, and communication regarding suspension, termination, accidents, spills, and exposures. The Relying Institution has responsibility for elements related to their own staff and facilities, including training, facility inspections, and an occupational medicine program. There are a number of responsibilities that are shared between the collaborating institutions, including compliance requirements (e.g., reporting of incidents to NIH). Table 2 provides a further list of requirements for the reviewing and relying IBCs.

**Table 2.** Responsibilities of participating institutions and IBCs outlined in IBC agreement

Responsibility	Reviewing IBC/institution	Relying institution
Registering the IBC with the NIH	Maintain current IBC registration with NIH OSP, as appropriate Notify other Participating Institutions if registration with the NIH OSP is terminated	Maintain current IBC registration with NIH OSP, as appropriate Notify other Participating Institutions if registration with the NIH OSP is terminated
Permitting of the IBC with any local agencies	Maintain local municipality registration for recombinant or biological materials as applicable	Maintain local municipality registration for recombinant or biological materials as applicable
Study protocol review and approval	Perform review consistent with applicable laws, regulations, and guidance Determine whether health surveillance and immunizations are necessary	Report to Reviewing IBC special considerations that may impact the review process Right to review and comment, but not approve application
Procedural issues	Make IBC minutes available to Relying Institution Allow attendance of (nonvoting) representatives of Relying Institution to IBC meeting	Acknowledges that Reviewing IBC holds jurisdiction over review and IBC oversight of ceded Research If requested by Reviewing IBC, send a Relying IBC representative to the meeting to ensure adequate consideration of location conditions. Executing additional requirements imposed by Reviewing IBC (e.g., additional training, health screenings)
Notification	Notify Investigators and Relying Institution of review decisions Notify Investigators of renewal deadlines Notify Relying Institution of Suspension or Termination Submitting Annual Report to NIH and applicable local municipality	Notifying Biological Safety Officer (BSO), Occupational Health and other institutional personnel of information received from Reviewing IBC
Accidents, spills, and exposures	Upon receiving information regarding an accident, spill or exposure, and in consultation with PI and BSO, determine if notification to Relying Institution is required. Determine with Relying Institution if external reporting is necessary.	Determine with Reviewing IBC if external reporting is necessary.
Noncompliance	Investigate allegations of noncompliance Afford Relying Institution an opportunity to attend any meetings associated with the investigation of noncompliance Notify Relying Institution of decision and steps necessary for remediation of noncompliance	Notify reviewing IBC of any identification of noncompliance
Inspections	Perform routine inspection of their own facilities As needed, perform routine, not-for-cause audits with reasonable notification of Relying Institution facilities.	Perform routine inspections of their own facilities. As needed, perform routine, not-for-cause audits with reasonable notification of Reviewing Institution facilities.
Training	Provide training to their respective Investigators, Research Personnel, and IBC members	Provide training to their respective Investigators, Research Personnel, and IBC members
Occupational Health and Safety	Provide an Occupational Health and Safety Program Agree to work with counterpart at other Participating Institutions where necessary	Provide an Occupational Health and Safety Program Agree to work with counterpart at other Participating Institutions where necessary
Internal injury reporting	Develop policies and procedures to address injuries, accidents, illnesses, or emergency situations Share these policies with Participating Institutions	Develop policies and procedures to address injuries, accidents, illnesses, or emergency situations Share these policies with Participating Institutions Report injuries, accidents, and serious adverse events to the Reviewing IBC
External reporting (e.g., NIH OSP, Municipalities, Sponsors)	Notify Relying Institution of determination that external reporting is needed. Give Relying Institution an opportunity to review and comment on report Consider filing a joint report with the Relying Institution	Notify Reviewing IBC of determination that external reporting is needed. Give Reviewing IBC an opportunity to review and comment on report Consider filing a joint report with the Reviewing IBC
Administrative reporting	Distribute relevant annual reports and/or meeting materials to Relying Institution (e.g., meeting minutes)	Distribute relevant annual reports to Reviewing IBC (e.g., safety reports)
Disagreements	Agree to work collaboratively to settle any differences. If a disagreement cannot be resolved, Participating Institutions may request that the respective Institutional Officials communicate to resolve the matter.	Agree to work collaboratively to settle any differences. If a disagreement cannot be resolved, Participating Institutions may request that the respective Institutional Officials communicate to resolve the matter.

IBC, Institutional Biosafety Committee; NIH, National Institutes of Health; OSP, Office of Science Policy; BSO, Biological Safety Officer; PI, Principal Investigator

To avoid confusion and to help standardize processes, model forms and other resources for ceding review have been developed [14]. In addition, the signatory institutions agreed to periodic review and re-evaluation of the sIBC agreement in order to allow for interval quality and operational improvement. The process is simplified by the development of and utilization of shared forms and server access.

### Examples of sIBC Agreement Deployment

There are a number of setting in which reliance on a sIBC is beneficial. Initially, the sIBC Agreement was developed to consolidate review of clinical gene-transfer studies that were conducted at multiple institutions in Boston and Cambridge. Many of these studies were oncology studies that underwent sIRB review but resulted in three or more IBC reviews. The sIBC Agreement now allows for sIBC review with the other institutions permitted to cede review. The sIBC Agreement is also useful in settings wherein core facilities are utilized by investigators from multiple institutions. For instance, an investigator may wish to conduct rDNA research requiring biosafety level 3 (BSL3) practices in a BSL3 core facility at a neighboring institution. While the investigator’s institution is obligated to review the proposed research, the institution that houses the BSL3 laboratory has the appropriate expertise on their IBC to conduct the review. The IBC Agreement allows for the IBC of the appropriate institution to conduct the review, while the second institution may rely. Additionally, sIBC is useful for experiments in which animals housed in one institution are exposed in vivo to rDNA (e.g., viral vector) and later require specialized imaging at a second institution’s imaging facility. The IBC Agreement allows the second institution’s IBC to rely on the review of first institution. Nevertheless, the biosafety officers at each institution must collaborate to ensure that all biosafety risks are managed. Finally, sIBC review has been utilized for research conducted at an institution without an existing IBC. The IBC Agreement allows one institution to rely on another institution’s IBC – even if that institution is not engaged in the research – so long as the reviewing IBC is properly constituted to address the requirements of the first institution (i.e., unaffiliated members are from the appropriate locale).

## Discussion

Developing and managing the IBC Agreement has led to several benefits. First, the initial frequency of meetings and discussion led to the development of trust among the IBC staff from different institutions; these relationships resulted in shared learning and rendered IBC operations and compliance concerns easier to manage in the future. Second, as anticipated, duplicative IBC review decreased. Third, easing the process of multisite research appeared to increase collaboration among investigators as well as more efficient access to core and/or specialized facilities. Finally, while difficult to quantify, we believe that investigator burden decreased.

The development of the Master IBC Reliance Agreement mirrors recent federal efforts to streamline approval processes for gene therapy research. The US FDA Center for Biologics Evaluation and Research streamlined the requirements for preclinical testing through product development and manufacturing [15], and in August 2018, NIH and FDA proposed changes to eliminate duplicative reporting requirements as well as review by the Recombinant DNA Advisory Committee [16]. Notably, however, the proposed changes did not address duplicative review by the IBCs themselves nor did it address institutional reliance in IBC review [17]. This current model, similar to that of sIRB review for multisite research, was developed to reduce administrative burden without compromising quality of review or oversight responsibilities.

The major challenges to the utilization of the Agreement included PI outreach and communication, perceived timeliness of review, trust, and PI understanding of changes to their responsibilities. The Harvard Catalyst IBC Working Group engaged with PIs to increase awareness of the availability of the Agreement. While we anticipated that the IBC Reliance Agreement would be utilized across all Harvard Catalyst institutions in the first year following acceptance, fewer studies than expected utilized reliance. The vast majority of protocols reviewed by the academic institutions are basic and translational research, where protocol amendments, and not new protocols, are utilized. For clinical research, one investigator will often initiate a study – sometimes months before new sites are added and only then must the Reviewing IBC coordinate the additional sites to streamline the approval. Developing communication tools have expanded the use of the agreement.

For success to be realized, the Reviewing IBC must assume responsibility for personnel not residing at their own institution. There have been no adverse event reporting, compliance, or audits issue to date, but these will inevitably arise. Research compliance reports, such as laboratory inspection and training, have been shared among IBCs through each institutional BSO, each of whom understand the confidentiality of these communications. As this model agreement extends to institutions that are less familiar with one another, we anticipate that confidentiality provisions may need to be documented.

### Scaling the Master IBC Agreement

The IBC Agreement provides a flexible and replicable framework for IBC reliance that can be adapted by other academic medical centers and research institutions. Limiting participation to local institutions ensures compliance with both federal and local municipality requirements in that unaffiliated community members are present to represent the interests of the surrounding community. The IBC Agreement does not prescribe reliance for all protocols but rather allows independent institutions to determine the appropriateness of reliance on a protocol-by-protocol basis. The roles and responsibilities of Reviewing IBCs and Relying Institutions have been outlined, and common processes are supported by tools and resources that promote compliance. In addition, ongoing efforts to harmonize processes will help to ensure compliance, assist with training, and standardize responsibilities of all staff involved. The IBC Agreement serves as an opportunity for ongoing program review. The potential to replicate or, if necessary, adapt this framework will help to decrease administrative burden, retain, and expand the authority of the IBC overseeing the research, and maintain protections of individuals and animals involved in the research.

Specific challenges regarding the federal and local requirements for unaffiliated community members currently limit further expansion of the utility of the agreement. We suggest that the NIH OSP consider revising the NIH Guidelines to allow for unaffiliated members to represent the concerns of communities more broadly, not restricted in their domain of responsibility to a specific and local geography. Such a modification would be similar to federal regulations governing human subject research [18] and could be considered at least for projects that do not involve facility construction or renovation, agents of bioterrorism [19], or agents contained at biosafety level 4. We believe that an unaffiliated community member is able to represent the sensitivities of communities and the public between and among geographies. Alternatively, a “local community member” could be included in the IBC review as an additional (and virtual) *ad hoc* member, not contributing to quorum considerations, thus maintaining the community representation requirement. We believe that the NIH Guidelines can be amended or interpreted (via guidance) to allow for the interests of the surrounding community to be adequately represented by either of these approaches. As a flexible and replicable framework, the IBC Reliance Agreement may serve as a proof-of-concept prototype to streamline IBC review and enable accelerated collaborative research among institutions.

## Footnotes

1 NIH has codified a requirement for sIRB review of multisite nonexempt human subjects research for all studies funded by the federal government.
